# Cardiac Magnetic Resonance Radiomics for Diagnosis, Phenotyping and Risk Stratification of Cardiomyopathies

**DOI:** 10.3390/healthcare14183104

**Published:** 2026-09-20

**Authors:** Cosimo Granitto, Kristi Hoxha, Gianmarco Forasassi, Giovanni Scribano, Alberto Cossu, Simona Tassinari, Simone Boldrin, Rita Pavasini, Federico Marchini, Gianluca Campo, Luigi Manco, Elisabetta Tonet

**Affiliations:** 1 Cardiology Unit, Azienda Ospedaliero Universitaria of Ferrara, Via Aldo Moro 8, Cona, 44124 Ferrara, Italykristi98hoxha@gmail.com (K.H.); pvsrti@unife.it (R.P.); mrcfrc2@unife.it (F.M.);; 2 Medical Physics Unit, University Hospital of Ferrara, 44124 Ferrara, Italy; gscribano.ext@ospfe.it (G.S.);; 3 Department of Radiology and Laboratory Medicine, University Hospital of Ferrara, 44121 Ferrara, Italy

**Keywords:** radiomics, cardiac magnetic resonance, cardiomyopathies

## Abstract

Background: Cardiovascular magnetic resonance (CMR) is the reference non-invasive imaging modality for evaluating cardiomyopathies, providing comprehensive assessment of cardiac morphology, function, and tissue characterization. Radiomics has recently emerged as an advanced image analysis technique that extracts quantitative imaging biomarkers from routine CMR images, potentially enhancing disease characterization beyond conventional visual assessment. This systematic review summarizes current evidence on the role of CMR-based radiomics in the diagnosis, phenotypic characterization and risk stratification of cardiomyopathies. Methods: A structured search identified English-language, peer-reviewed studies published up to August 2026 investigating CMR-based radiomics in hypertrophic, dilated, arrhythmogenic, and infiltrative cardiomyopathies, including cardiac amyloidosis, Fabry disease, and cardiac sarcoidosis. Study selection followed PRISMA 2020 guidelines. Methodological quality was assessed using the Methodological Index for Non-Randomized Studies (MINORS) and the Radiomics Quality Score 2.0 (RQS 2.0) The authors have reviewed and edited the output and take full responsibility for the content of this publication. Results: Current evidence indicates that CMR radiomics provides incremental diagnostic information beyond conventional CMR by quantifying myocardial tissue heterogeneity. Radiomic features derived from cine imaging, late gadolinium enhancement, native T1/T2 mapping, and extracellular volume maps showed promising performance in differentiating cardiomyopathy subtypes, distinguishing pathological from physiological remodeling, and identifying infiltrative and inflammatory myocardial diseases. Multiparametric radiomic models generally outperformed individual imaging biomarkers, with preliminary evidence supporting applications in risk stratification and outcome prediction. However, studies were predominantly retrospective, involved small cohorts, used heterogeneous imaging and radiomics workflows, and rarely included external validation. RQS 2.0 assessment demonstrated low-to-moderate methodological quality. Conclusions: CMR-based radiomics shows considerable potential for improving diagnosis and phenotypic characterization of cardiomyopathies. However, methodological standardization, multicenter prospective validation, and demonstration of incremental clinical value are required before routine clinical implementation.

## 1. Introduction

Cardiomyopathies represent a heterogeneous group of myocardial disorders associated with significant morbidity, mortality, and risk of sudden cardiac death. Accurate diagnosis and phenotypic characterization are essential for patient management, risk stratification, and therapeutic decision-making. In this context, cardiovascular magnetic resonance (CMR) has emerged as the reference non-invasive imaging modality for the assessment of cardiomyopathies, owing to its superior spatial resolution and its unique ability to provide comprehensive information on cardiac morphology, function, tissue composition, and myocardial fibrosis through multiparametric techniques. Beyond conventional CMR parameters—such as ventricular volumes, ejection fraction, late gadolinium enhancement (LGE), and parametric mapping—there is growing interest in advanced image analysis methods. Radiomics, originally formalized by Lambin et al., refers to the high-throughput extraction of quantitative features describing myocardial intensity, texture, shape, and spatial heterogeneity, potentially capturing subtle tissue characteristics that are imperceptible to the human eye [1].

In this perspective, medical images are not only visual representations but structured quantitative data that may support diagnostic, prognostic, and predictive modelling. In recent years, an increasing number of studies have investigated the application of CMR-based radiomics in various cardiomyopathies, including hypertrophic, dilated, arrhythmogenic, and infiltrative forms such as cardiac amyloidosis. These studies have explored the ability of radiomic features derived from cine images, LGE sequences, and parametric maps to differentiate between cardiomyopathy subtypes, distinguish pathological from physiological remodeling, and, in some cases, predict clinical outcomes. Despite promising preliminary results, the evidence remains fragmented, with substantial heterogeneity in imaging protocols, segmentation strategies, feature extraction methods, machine learning algorithms, and validation approaches. Given these methodological challenges, the Radiomics Quality Score 2.0 may provide a structured framework to assess the reproducibility, robustness, and clinical translation potential of published CMR radiomics studies [2]. The aim of this systematic review is to evaluate the current evidence on the use of CMR-based radiomics for the diagnosis, phenotypic characterization, differential diagnosis and risk stratification of cardiomyopathies. Specifically, we sought to summarize the types of cardiomyopathies investigated, imaging sequences and radiomic approaches employed, machine learning models applied, and diagnostic and prognostic performance metrics reported, while assessing methodological quality and highlighting areas for future research.

## 2. Search Strategy and Eligibility Criteria

A structured search of full-text articles published in English until August 2026 was performed. The search strategy included the following terms: (radiomics OR “texture analysis” OR “radiomic features”) AND ((CMR) OR (cardiac magnetic resonance)). Eligible studies included original articles evaluating radiomic features extracted from CMR images in patients with amyloidosis, Fabry disease, hypertrophic cardiomyopathy (HCM), idiopathic dilated cardiomyopathy (IDCM), arrhythmogenic cardiomyopathy and sarcoidosis. The databases analyzed were PubMEd, Embase, Web of Science, Scopus, Biomed Central, and Cochrane Library. Only papers published in English and in peer-reviewed journals were selected. The inclusion criteria of the studies were: observational or clinical trials investigating radiomics in CMR. The review protocol was prospectively registered in the Open Science Framework (OSF; DOI: 10.17605/OSF.IO/AF82N).

**Figure 1:**
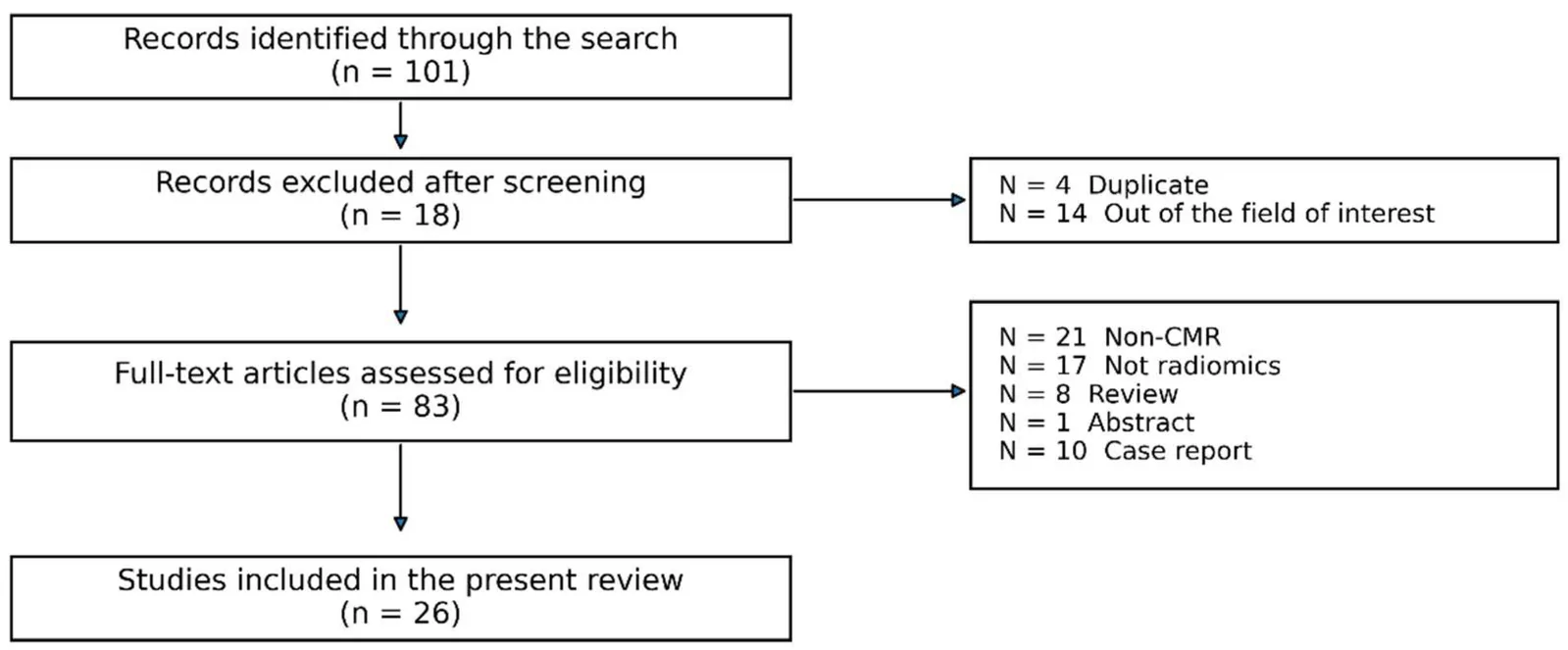
PRISMA 2020 flow diagram of study selection.

Overall, 101 records were identified. After evaluation of title and abstract, a total of 83 studies were analyzed as full text. The general methodological quality of selected papers was tested using Methodological Index for Non-Randomized Studies (MINORS) criteria [3]. Unblinded reviewers performed the analysis of the full texts for quality assessment. Discrepancies between reviewers have been solved by consensus. The maximum score obtained was 14 and the minimum 8. We included in the present review only studies with a score ≥10 in order to exclude studies with lower overall methodological quality. A total of 26 papers were included in the final review, as summarized in the PRISMA 2020 flow diagram [4] (Figure 1).

For each included study, radiomics-specific methodological characteristics were extracted, including CMR sequence, region of interest and segmentation approach, feature extraction method, feature selection strategy, model development, validation approach, and availability of external validation or open science elements. These data were summarized in Table 1.

Radiomics-specific methodological quality was assessed using the Radiomics Quality Score 2.0 (RQS 2.0). Given that the included studies were based on handcrafted radiomics and were predominantly exploratory or preclinical, RQS 2.0 was applied using the handcrafted radiomics branch up to Radiomics Readiness Level (RRL) 6, with a maximum achievable score of 39 points. For each study, the final score was reported both as an absolute value and as a percentage of the maximum achievable score. Scoring was performed independently by two reviewers, and discrepancies were resolved by consensus [2]. RQS 2.0 was used as a descriptive quality measure and was not applied as an exclusion criterion. During the preparation of this manuscript, the authors used ChatGPT (Open AI) for the purposes of editing only.

## 3. Radiomics: Principles

**Figure 2:**
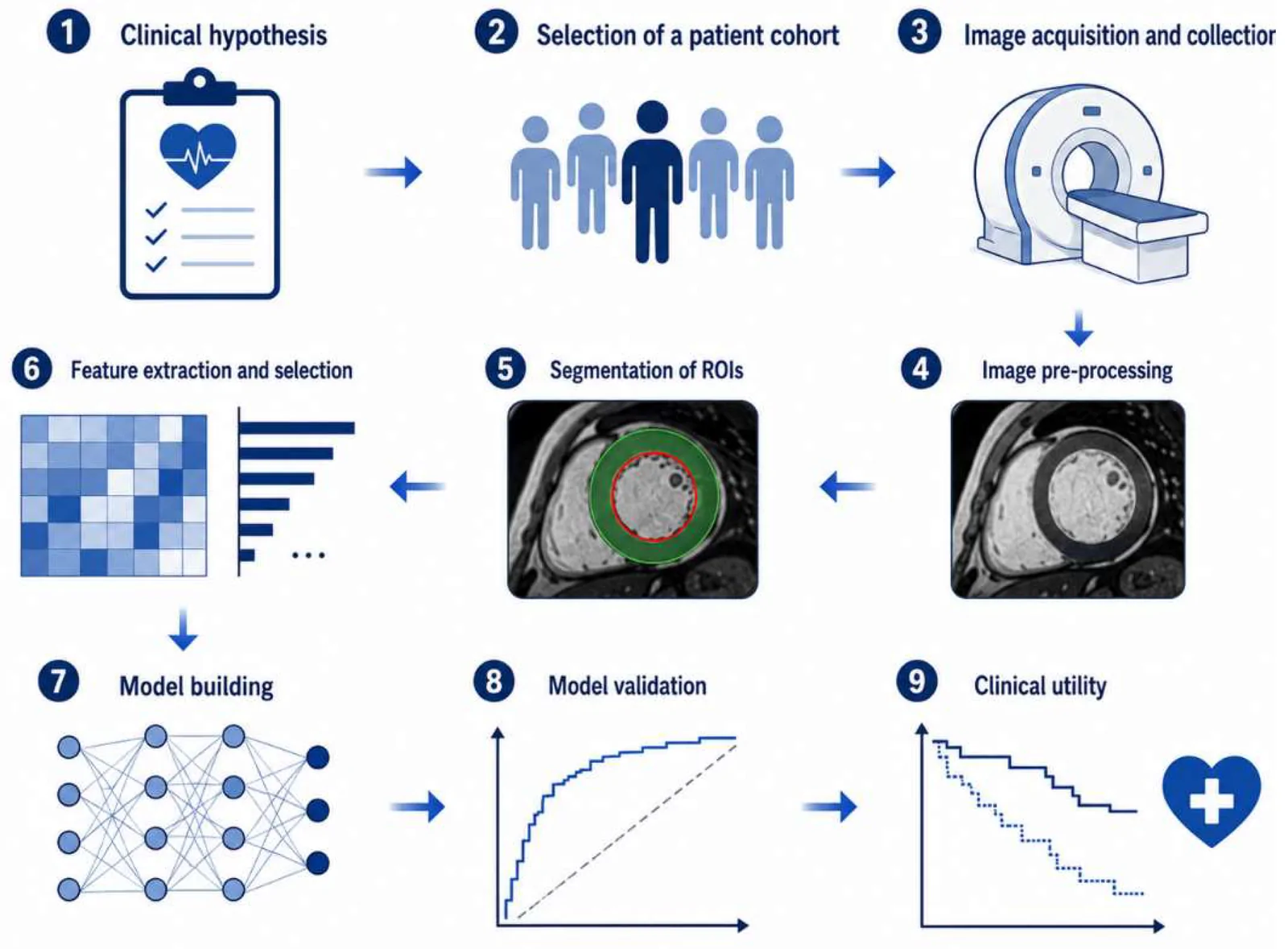
Schematic overview of the CMR radiomics workflow.

Radiomics is an image analysis approach that aims to convert medical images into high-dimensional quantitative data by extracting a large number of predefined features that characterize tissue intensity, texture, shape, and spatial heterogeneity. The underlying assumption of radiomics is that medical images contain information reflecting pathophysiological processes at the microstructural and molecular level, which may not be appreciable by visual assessment alone. In CMR, the radiomics workflow starts from a clinically relevant hypothesis and the selection of an appropriate patient cohort, followed by standardized image acquisition, image pre-processing, segmentation of cardiac regions of interest, feature extraction and selection, model building, model validation, and assessment of clinical utility (Figure 2). Extracted features are commonly grouped into three main categories. First-order features describe the distribution of voxel intensities within the segmented region without considering their spatial relationships. Typical examples are mean intensity, variance, skewness, and kurtosis, which provide information on signal distribution, variability, asymmetry, and distributional shape. Shape-based features describe the geometry of the segmented region and include volume, surface area, and sphericity. Texture features characterize how voxel intensities are spatially arranged within the tissue. They therefore provide information on whether an image region is relatively homogeneous or contains complex spatial variations. Texture features are commonly derived from matrices such as the gray-level co-occurrence matrix (GLCM), gray-level run-length matrix (GLRLM), and gray-level size-zone matrix (GLSZM). These matrices describe relationships between neighbouring intensity values, runs of similar intensities, and the size of homogeneous regions, respectively. Texture features may therefore quantify tissue heterogeneity that is not readily appreciable by visual assessment alone. Radiomic features can also be extracted from filtered or transformed images. Wavelet decomposition and Laplacian-of-Gaussian filtering, for example, can emphasize image patterns at different spatial scales and reveal additional information on tissue heterogeneity.

Given the large number of extracted features and the relatively limited sample sizes typical of medical imaging studies, feature selection and dimensionality reduction are critical steps to reduce redundancy and mitigate overfitting. Selected features can subsequently be incorporated into statistical or machine-learning models for specific tasks such as disease classification or risk stratification. Alternatively, deep-learning approaches may learn relevant image representations directly from the imaging data without requiring predefined handcrafted features. Model performance is usually evaluated using metrics such as accuracy, area under the receiver operating characteristic curve, sensitivity, and specificity, ideally with internal cross-validation and external validation cohorts. In the context of CMR, radiomics can be applied to multiple sequences, including cine imaging, late gadolinium enhancement (LGE), and parametric mapping, enabling a multiparametric characterization of myocardial tissue. However, radiomic features are sensitive to image acquisition, reconstruction, segmentation, preprocessing, intensity normalization, resampling, and gray-level discretization. Therefore, feature robustness, standardized feature definitions, transparent reporting, and external validation are essential for clinical translation. Initiatives such as the Image Biomarker Standardisation Initiative have been proposed to improve methodological consistency and reproducibility across software platforms and institutions, toward the implementation of radiomics in clinical practice [30].

## 4. Radiomics Workflow and Methodological Quality

Radiomics workflow characteristics and RQS 2.0 assessment are summarized in Table 1. Overall, the included studies used heterogeneous CMR inputs, including cine imaging, LGE, native T1 mapping, T2-weighted imaging, T2 mapping, ECV maps, and, in one study, combined PET and LGE-CMR data. Most studies relied on manually segmented myocardial regions of interest, whereas automated segmentation was reported only in selected studies. Feature extraction was mainly based on handcrafted texture or radiomic features, using dedicated radiomics software or custom pipelines. Feature selection and machine-learning modelling were commonly performed, most often using LASSO-based methods, recursive feature elimination, principal component analysis, support vector machines, random forests, logistic regression, or ensemble learning approaches. Internal validation strategies, including cross-validation or train-test splitting, were frequently reported, whereas independent external validation was available only in a minority of studies. Accordingly, RQS 2.0 scores were generally low to moderate, ranging from 5/39 (13%) to 22/39 (56%), reflecting the exploratory nature of most available evidence and the limited degree of clinical translation readiness.

## 5. Dilated Cardiomyopathy

CMR radiomics is emerging as a promising tool for the evaluation of dilated cardiomyopathy (DCM), as it may capture myocardial heterogeneity beyond conventional volumetric, functional, and tissue characterization parameters. On conventional CMR, non-ischemic DCM is typically characterized by left ventricular dilatation, global systolic dysfunction, increased native T1 and extracellular volume (ECV) consistent with diffuse interstitial fibrosis, and, when present, a non-ischemic mid-wall pattern of LGE, most commonly involving the interventricular septum. Against this background, radiomics may provide incremental value by extracting high-dimensional quantitative information from routinely acquired CMR sequences, including cine imaging, native T1 mapping, ECV, and LGE, thereby capturing subtle variations in myocardial texture and spatial heterogeneity that are not fully appreciable by visual assessment alone [18,31,32]. Initial evidence in DCM has mainly derived from studies based on texture analysis of parametric mapping. Texture features extracted from native T1 mapping have been shown to discriminate patients with DCM from healthy controls, supporting the concept that myocardial abnormalities in DCM are not fully captured by mean T1 values alone [3]. Likewise, texture analysis of native T1 maps has been reported to characterize diffuse interstitial fibrosis patterns and to provide incremental information beyond average native T1 measurements, suggesting that radiomics may better reflect the spatial complexity of fibrotic remodeling in DCM [7]. More recently, radiomic signatures derived from native T1, ECV, and LGE images in non-ischemic DCM have been associated with histological myocardial composition on endomyocardial biopsy, further supporting the potential of radiomics as a non-invasive surrogate of myocardial microstructural remodeling [18]. Beyond tissue characterization, CMR radiomics has also shown potential for differential diagnosis in patients with overlapping cardiomyopathic phenotypes. Radiomics features extracted from CMR images have been reported to discriminate among DCM, hypertrophic cardiomyopathy, and healthy controls [16], while radiomics signatures derived from short-axis CMR images have also demonstrated high diagnostic performance in distinguishing left ventricular non-compaction, hypertrophic cardiomyopathy, and DCM, even without dedicated trabecular delineation [20]. These findings suggest that radiomics may improve phenotypic classification when conventional morphologic interpretation is inconclusive [16,20]. A particularly relevant application is the differentiation between ischemic cardiomyopathy and DCM. Machine-learning models based on cine-CMR radiomics, especially myocardial features, have shown the ability to distinguish ischemic from dilated cardiomyopathy and may outperform models based on clinical variables alone [19]. This appears biologically plausible, since ischemic cardiomyopathy is typically characterized by regional scar-related remodeling, whereas DCM more often reflects global ventricular remodeling and diffuse interstitial myocardial abnormalities. Radiomics may therefore capture differences in myocardial texture, spatial heterogeneity, and cavity-myocardium relationships that mirror these distinct substrates. Consistently, radiomics derived from non-contrast cine CMR has also demonstrated the ability to differentiate ischemic and dilated cardiomyopathy while identifying myocardial scar, highlighting a potentially valuable strategy in patients with contraindications to gadolinium administration [21]. In a broader heart-failure population, multiparametric non-contrast CMR combining cine imaging, native T1/T2 mapping, and texture analysis has also shown potential for identifying different etiologies of heart failure, with possible relevance for dilated phenotypes [17]. Overall, the available evidence supports the feasibility of CMR radiomics in DCM for disease detection, tissue characterization, and differential diagnosis (Table 2 and Table 3). However, current studies remain limited by relatively small sample sizes, frequent single-center designs, heterogeneity in acquisition and segmentation workflows, instability of radiomic features, and limited external validation [31,32]. Although the field is also beginning to move toward prognostic applications [33], larger multicenter studies and standardized pipelines will be necessary before CMR radiomics can be integrated into routine clinical assessment of DCM [18,31,32].

## 6. Hypertrophic Cardiomyopathy

The central role of CMR in the setting of hypertrophic cardiomyopathy (HCM) has been previously demonstrated, providing relevant diagnostic and prognostic information (Table 2). Radiomics offers a quantitative framework to extract texture and heterogeneity features from standard CMR images [5]. On one hand, radiomics could be useful for differential diagnosis. Regarding HCM versus hypertensive heart disease (HHD), multiple studies have shown that radiomic models outperform conventional CMR parameters. Using native T1 mapping, Neisius et al. demonstrated that selected texture descriptors discriminated HCM from HHD more accurately than global native T1 values, even in patients with similar wall thickness, highlighting the ability of radiomics to capture the focal, heterogeneous fibrosis typical of HCM as opposed to the more diffuse remodeling of HHD [9,10].

Expanding this approach, Liu et al. incorporated papillary muscle-derived features into a machine learning model, significantly improving discrimination and underscoring the contribution of subtle geometric remodeling patterns beyond myocardial parenchyma alone [8]. In a larger cohort, Zhang et al. integrated cine- and LGE-derived radiomic scores with conventional parameters, achieving superior diagnostic performance compared with clinical models, confirming the incremental value of multiparametric radiomic signatures [14]. On the other hand, radiomics has demonstrated the ability to discriminate between MYH7- and MYBPC3-related HCM despite similar conventional CMR metrics, indicating the presence of genotype-specific microstructural signatures not appreciable by standard assessment [9].

Beyond diagnostic applications, radiomics may also contribute to prognostic risk stratification in HCM. In a large cohort study, Wang et al. demonstrated that radiomic features extracted from LGE images reflecting myocardial scar heterogeneity were associated with the risk of sudden cardiac death, suggesting that texture-derived metrics may provide incremental information for identifying high-risk patients beyond conventional clinical and imaging parameters [12,13].

## 7. Amyloidosis

Cardiac amyloidosis (CA) is an infiltrative cardiomyopathy characterized by extracellular deposition of misfolded proteins within the myocardium, leading to progressive diastolic dysfunction, restrictive physiology, and progressive heart failure with adverse outcomes. The phenotypic overlap with other causes of left ventricular hypertrophy and underestimation of early infiltration remains a significant diagnostic challenge in this context. Radiomics has emerged as a promising approach to extract high-dimensional quantitative features from CMR images, capturing subtle microstructural alterations beyond visual assessment and potentially improving differential diagnosis and disease characterization. Schofield et al. demonstrated that texture analysis of balanced steady-state free precession (bSSFP) cine images could differentiate among various etiologies of left ventricular hypertrophy. Parameters such as kurtosis and skewness showed consistent differences across disease groups and healthy controls, with acceptable test–retest reproducibility, supporting the technical robustness of cine-based radiomic analysis [11]. Similarly, Jiang et al. showed that machine learning-based radiomics applied to non-contrast cine images accurately discriminates CA from HCM, with gray-level non-uniformity (GLevNonU) emerging as a key descriptor of myocardial microstructural differences. In this study the combination of radiomic features with conventional MR metrics improved discrimination compared with conventional metrics alone, suggesting incremental diagnostic value. Moreover, the association between GLevNonU and LGE positivity indicates that cine-derived heterogeneity metrics may reflect underlying myocardial infiltration or fibrosis [23]. Parametric mapping-based radiomics has further refined non-contrast evaluation. Texture analysis of T2-weighted imaging has shown feasibility in differentiating CA from HCM. Huang et al. evaluated texture features derived from T2-weighted CMR in a large cohort of CA and HCM patients and identified seven independent features that provided strong discriminatory power. The diagnostic performance of the texture model was comparable to that of LGE quantification, supporting the hypothesis that T2-based radiomics may capture microenvironmental alterations, such as changes in water content and interstitial expansion, that are not fully appreciated by conventional assessment [22]. Analyzing T1 mapping, Antonopoulos et al. extracted hundreds of radiomic features from T1 maps and demonstrated that a subset of six features outperformed mean native T1 values in distinguishing myocardial health from disease and in differentiating HCM phenocopies, including cardiac amyloidosis. In particular, the radiomics model achieved superior AUCs compared with T1 values alone across multiple disease categories [6]. More specifically in AL-CA, Ma et al. showed that a radiomics score derived from native T1 mapping provided higher sensitivity for detecting myocardial involvement than native T1 values. Furthermore, a combined model incorporating T1 values and a selected radiomic feature further improved diagnostic performance [24]. These findings are clinically relevant, as native T1-based radiomics may represent a contrast-free alternative to ECV quantification, particularly advantageous in patients with renal impairment or contraindications to gadolinium administration. More recently, multiparametric and ensemble machine learning approaches have been proposed to enhance diagnostic accuracy. In a single-center retrospective study, Zhang et al. developed an ensemble model integrating radiomic features from native T1, post-contrast T1, T2 mapping, and ECV, achieving excellent multiclass discrimination among AL-CA, HCM and healthy controls. Texture and first-order features from mapping sequences dominated the model, while ventricular shape features played only a marginal role, reinforcing the concept that microstructural tissue alterations rather than geometric remodeling drive discrimination between these entities [15]. However, radiomics applied to LGE-CMR has also provided incremental diagnostic value. In a multicenter study including biopsy-proven AL amyloidosis, Zhou et al. demonstrated that radiomic signatures extracted from LGE images achieved superior diagnostic performance compared with visual assessment and conventional quantitative LGE metrics, with basal-segment features providing the highest discriminatory power. Moreover, radiomic scores showed a moderate correlation with Mayo stage, suggesting that LGE-derived radiomics may reflect not only the presence but also the severity of myocardial involvement. These findings raise the possibility that radiomics could contribute to prognostic stratification in cardiac amyloidosis, although prospective validation is required [25].

## 8. Other Cardiomyopathies

Among other cardiomyopathies, the application of CMR radiomics remains more limited, but available evidence suggests a potential role in phenotyping, disease activity assessment, and risk stratification. In arrhythmogenic right ventricular cardiomyopathy (ARVC), recent work has shown that radiomics derived from pericardial fat tissue on CMR can predict left ventricular involvement and provide incremental prognostic information for major adverse cardiovascular events. This is particularly interesting, as ARVC is increasingly recognized as a biventricular disease, and radiomics may capture subtle structural and tissue-related changes extending beyond conventional functional parameters alone [26].

In cardiac sarcoidosis, CMR-based radiomics appears especially promising for refining tissue characterization and disease activity assessment. Radiomic analysis of LGE images has been shown to distinguish active from inactive cardiac sarcoidosis, using PET-CMR-defined inflammatory activity as a reference standard, suggesting that radiomics may capture textural signatures associated with ongoing myocardial inflammation beyond the visual presence of scar alone [28]. In addition, radiomic features derived from LGE-CMR have also been investigated to differentiate cardiac sarcoidosis from other inflammatory myocardial conditions, including post-COVID myocardial inflammation, supporting the potential of advanced image analysis to improve differential diagnosis in clinically challenging settings [29]. A further quantitative LGE-CMR study comparing cardiac sarcoidosis with other non-ischemic cardiomyopathy phenotypes showed that sarcoidosis is characterized by higher and more regionally concentrated LGE signal intensity, particularly within the septum and along the right-sided endocardial layer, reinforcing the concept that the pattern and distribution of myocardial injury in sarcoidosis differ substantially from the more diffuse interstitial remodeling typically observed in non-ischemic dilated cardiomyopathy [27,34].

In Fabry disease, CMR plays a central role in cardiac phenotyping, with characteristic findings including low native T1 values related to sphingolipid storage, possible pseudonormalization in more advanced inflammatory stages, and inferolateral mid-wall LGE. However, dedicated peer-reviewed evidence specifically focused on CMR radiomics in Fabry cardiomyopathy remains scarce. Therefore, although advanced image analysis may represent a promising future direction for differentiating Fabry cardiomyopathy from phenotypically overlapping conditions such as hypertrophic cardiomyopathy, robust radiomics-based validation is still lacking [35].

Overall, current evidence suggests that in ARVC and cardiac sarcoidosis, CMR radiomics may provide incremental value for phenotyping, risk assessment, and differential diagnosis, whereas in Fabry disease the field remains largely exploratory. These findings further emphasize both the promise of radiomics across less common cardiomyopathies and the need for larger, standardized, multicenter studies before clinical implementation can be considered.

## 9. Conclusions and Future Directions

CMR radiomics represents a promising extension of conventional CMR, enabling the extraction of quantitative imaging biomarkers that capture myocardial tissue heterogeneity beyond visual assessment and standard functional or tissue characterization parameters. Across the spectrum of cardiomyopathies, including hypertrophic, dilated, infiltrative, inflammatory, and arrhythmogenic diseases, radiomics has consistently demonstrated the potential to improve diagnostic accuracy, facilitate differential diagnosis, and refine disease phenotyping. Emerging evidence also suggests a possible role in prognostic assessment, although this remains less well established than its diagnostic applications. Despite these encouraging results, current evidence is still limited by relatively small retrospective cohorts, methodological heterogeneity, variability in image acquisition and segmentation, differences in radiomic feature extraction and machine learning pipelines, and the scarcity of external validation. Consequently, the incremental clinical value of radiomics beyond established CMR biomarkers has yet to be definitively demonstrated. At present, CMR radiomics should therefore be regarded as a promising research tool rather than a technique ready for routine clinical implementation. The RQS 2.0 assessment further supports the need for methodological strengthening in this field. Although most studies described image inputs, segmentation strategies, feature extraction, and model development, RQS 2.0 scores were generally low to moderate. The highest scores were observed in studies incorporating external validation, automated segmentation, prognostic follow-up, calibration analysis, or open science elements. Conversely, limited external validation, small retrospective cohorts, manual segmentation, heterogeneous preprocessing, and incomplete reporting of robustness, reproducibility, and clinical utility were common limitations. These findings indicate that current CMR radiomics evidence in cardiomyopathies remains largely exploratory, with insufficient clinical translation readiness for routine implementation. Future research should focus on improving the robustness, reproducibility, and clinical applicability of CMR radiomics. Large prospective multicenter studies with standardized acquisition protocols, harmonized image preprocessing, and adherence to international standardization initiatives will be essential to ensure reproducible radiomic biomarkers across scanners, vendors, and institutions. Equally important is the adoption of transparent reporting standards and rigorous external validation to improve the generalizability of predictive models. Another important direction will be the integration of radiomics with complementary sources of information, including clinical variables, genetic data, circulating biomarkers, and conventional CMR parameters, to develop multimodal precision medicine models capable of supporting individualized diagnosis and risk stratification. Advances in artificial intelligence, particularly deep learning and hybrid radiomics–deep learning approaches, may further enhance model performance while reducing the need for handcrafted feature engineering. In parallel, the development of fully automated segmentation and analysis pipelines could facilitate workflow integration and improve reproducibility in everyday clinical practice. Ultimately, the clinical success of CMR radiomics will depend not only on achieving high diagnostic accuracy but also on demonstrating meaningful incremental value over existing imaging biomarkers, improving clinical decision-making, and positively influencing patient outcomes through prospective clinical validation studies.

## Figures and Tables

**Table 1:** Radiomics workflow and methodological quality of included CMR radiomics studies.

First Author, Year	Imaging Data	ROI/Segmentation	Feature Extraction	Feature Selection/AI Model	Validation	RQS 2.0 Score *
Amano et al., 2020 [5]	T2-STIR	Hypertrophied myocardial regions and control septum; manual ROI	Texture analysis with MaZda	ROC-based feature evaluation	No independent validation	5/39 (13%)
Antonopoulos et al., 2021 [6]	Native T1 mapping	LV myocardium on basal short-axis slice; manual segmentation	3D Slicer; 850 radiomic features	PCA, unsupervised clustering, and RF-based feature selection	Internal cross-validation and test set	9/39 (23%)
El-Rewaidy et al., 2020 [7]	Native T1 mapping	LV myocardium; manually segmented T1 maps transformed to standardized polar maps	Custom texture pipeline; 152 features	Sequential feature selection; SVM and regression models	70/30 split with cross-validation and test dataset	13/39 (33%)
Liu et al., 2023 [8]	Cine-CMR	MYO and PM; manual segmentation after image quality control	PyRadiomics; 3D radiomic features	Feature selection; SVM, LR, RF, KNN, and DT	70/30 train-test split with five-fold CV	10/39 (26%)
Neisius et al., 2019 [9]	Native T1 mapping	LV myocardium; manual segmentation	Texture descriptors from T1 maps	Sequential feature selection; SVM classifier	4:1 feature-selection/test-validation split	12/39 (31%)
Neisius et al., 2020 [10]	Native T1 mapping and LGE reference	LV myocardium on native T1 maps matched to LGE	Texture descriptors from native T1 images	Sequential feature selection; decision tree ensemble	Split-sample validation with independent test cohort	13/39 (33%)
Schofield et al., 2019 [11]	Cine bSSFP	Mid short-axis LV myocardium; manual ROI	TexRAD filtration-histogram texture analysis	Texture parameter comparison across LVH etiologies	Test-retest reproducibility cohort; no external validation	7/39 (18%)
Wang et al., 2020 [12]	Native T1 mapping	LV myocardium; manual segmentation	Radiomic feature extraction from T1 maps	PCA and SVM classifier	4:1 feature-selection/test-validation split	10/39 (26%)
Wang et al., 2021 [13]	LGE-CMR	LV myocardium/LGE images; manual delineation	157 LGE-derived radiomic features	CoxNet, LASSO, elastic net, and RF	Cross-validation; prognostic follow-up cohort	12/39 (31%)
Zhang et al., 2024 [14]	Cine-CMR and LGE-CMR	LV myocardium; segmentation on cine and LGE images	PyRadiomics; cine and LGE radscores	ICC filtering, correlation filtering, and logistic regression	6:4 train-validation split; calibration analysis	17/39 (44%)
Zhang et al., 2025 [15]	Native T1, post-contrast T1, T2 mapping, and ECV	Myocardial ROIs on parametric maps	PyRadiomics; multiparametric radiomic features	Multiple feature-selection methods and ML classifiers; ensemble soft-voting model	Development cohort and hold-out testing dataset	14/39 (36%)
Zhang et al., 2023 [16]	Cine-CMR public datasets	LV, RV, and MYO segmentations from public datasets	PyRadiomics; features from ED and ES frames	Ten feature-selection methods and nine ML classifiers	Test dataset with cross-validated performance	14/39 (36%)
Amano et al., 2025 [17]	Cine-CMR, native T1/T2 mapping	Mid-septal myocardial segment; manual ROI	T1 texture analysis with open-access software	ROC-based assessment of texture and mapping parameters	No independent validation	6/39 (15%)
Amyar et al., 2025 [18]	Native T1, ECV, and LGE	Mid-septal myocardium near biopsy region; manual ROI	First-order and texture features from multiparametric CMR	Hierarchical clustering; medoid-based logistic regression	No external validation; histology used as reference	10/39 (26%)
Deng et al., 2024 [19]	Cine-CMR	LV, LVC, and MYO ROIs	Cine-derived radiomic features	LASSOCV/RFECV and ten ML classifiers	Five-fold CV and independent test set	12/39 (31%)
Izquierdo et al., 2021 [20]	Cine-CMR	LV short-axis myocardium and trabecular region; segmentation-based analysis	Shape, first-order, GLCM, GLRLM, and LBP features	SVM, LR, and RF one-vs-rest classifiers	Cross-validation; no external validation	11/39 (28%)
Lasode et al., 2025 [21]	Non-contrast cine-CMR	MYO and blood-pool ROIs; manual segmentation	Cine-derived radiomic features	ICC filtering, RFECV, and logistic regression	Training/test evaluation; comparison with radiologist performance	14/39 (36%)
Shao et al., 2018 [3]	Native T1 mapping	LV myocardium; manual endocardial and epicardial contours	Histogram and GLCM texture features	SVM classifier	Internal validation only	6/39 (15%)
Huang et al., 2022 [22]	T2-weighted CMR	Myocardial ROIs independently delineated by two radiologists	T2-weighted texture features	Reproducibility-based feature selection and ML modelling	7:3 train-test split; similar-hypertrophy subgroup validation	13/39 (33%)
Jiang et al., 2022 [23]	Non-contrast cine-CMR	LV myocardium on cine images; manual segmentation	Cine-derived radiomic features	ML-based feature selection and classification; combined CMR-radiomics model	70/30 train-validation split	12/39 (31%)
Ma et al., 2024 [24]	Native T1 mapping	Myocardial ROIs on native T1 maps	Native T1-derived radiomic features	LASSO logistic regression; combined T1-radiomics model	Training and test sets	12/39 (31%)
Zhou et al., 2022 [25]	LGE-CMR	Global LV myocardium and segmental LV ROIs	LGE-derived radiomic features	Feature selection and radiomics score modelling	Training, internal validation, and external multicohort validation	18/39 (46%)
Guo et al., 2025 [26]	Cine-CMR	PFT automatically segmented using 3SUnet	PyRadiomics; 851 PFT features	Correlation filtering, LASSO, logistic regression, and Cox models	Development set and external test set; prognostic follow-up	22/39 (56%)
Le et al., 2025 [27]	LGE-CMR	Myocardial LGE segmentations	PyRadiomics plus deep-learning-based MIL framework	L1-regularized feature selection; ABMIL classifier	Stratified five-fold CV; open images, segmentations, and code	20/39 (51%)
Mushari et al., 2023 [28]	LGE-CMR	LV myocardium; manual segmentation in 3D Slicer	PyRadiomics; 94 features	Logistic regression and PCA-based signatures	Stratified five-fold CV	10/39 (26%)
Mushari et al., 2024 [29]	PET and LGE-CMR	Whole LV ROIs on PET and LGE-CMR; manual segmentation in 3D Slicer	PyRadiomics; 94 PET/CMR features	Mann–Whitney testing, logistic regression, feature-retention thresholds	Stratified five-fold CV	11/39 (28%)

* RQS 2.0 score calculated using the handcrafted radiomics branch up to RRL 6, maximum score 39 points. Percentages were calculated as (obtained score/39) × 100. Abbreviations: ABMIL: attention-based multiple instance learning; bSSFP: balanced steady-state free precession; CMR: cardiovascular magnetic resonance; CV: cross-validation; DT: decision tree; ECV: extracellular volume; ED: end-diastole; ES: end-systole; GLCM: gray-level co-occurrence matrix; GLRLM: gray-level run-length matrix; ICC: intraclass correlation coefficient; KNN: k-nearest neighbor; LASSO: least absolute shrinkage and selection operator; LBP: local binary pattern; LGE: late gadolinium enhancement; LR: logistic regression; LV: left ventricular; LVC: left ventricular cavity; MIL: multiple instance learning; ML: machine learning; MYO: myocardium; PCA: principal component analysis; PET: positron emission tomography; PFT: pericardial fat tissue; PM: papillary muscle; RF: random forest; RFECV: recursive feature elimination with cross-validation; ROI: region of interest; RRL = Radiomics Readiness Level; RV: right ventricular; STIR: short tau inversion recovery; SVM: support vector machine.

**Table 2:** Clinical overview of CMR radiomics applications across cardiomyopathies.

Cardiomyopathy	Potential Role of Radiomics	Current Limitations
HYPERTROPHIC CARDIOMYOPATHY (HCM)	- Detection of subtle myocardial texture alterations not visible on conventional CMR; - Improvement in differentiation between HCM and other causes of left ventricular hypertrophy; - Identification of early myocardial changes even in patients without LGE.	- Most studies are retrospective with relatively small cohorts; - Lack of standardized radiomics pipelines and feature selection methods; - Limited external validation across scanners and institutions.
DILATED CARDIOMYOPATHY (DCM)	- Characterization of diffuse myocardial remodeling and fibrosis beyond conventional parameters; - Improvement in differentiation between ischemic and non-ischemic cardiomyopathy; - Radiomic features may correlate with histopathological findings such as fibrosis or inflammation.	- Radiomics signatures are heterogeneous and derived from different imaging sequences; - Many studies include mixed cardiomyopathy populations; - Clinical integration and prognostic validation remain limited.
CARDIAC AMYLOIDOSIS (CA)	- Improvement in non-invasive detection of myocardial infiltration; - Differentiation between cardiac amyloidosis and hypertrophic cardiomyopathy; - Texture features from cine, T1, T2, or LGE images capture microstructural changes associated with amyloid deposition.	- Complex machine learning algorithms reducing interpretability; - Small sample sizes and single-center designs; - Lack of standardized acquisition protocols and multicenter validation.
ARRHYTHMOGENIC RIGHT VENTRICULAR CARDIOMYOPATHY (ARVC)	- Radiomics of pericardial fat or myocardial tissue may provide additional information on disease phenotype; - Prediction of left ventricular involvement and adverse outcomes; - Potential improvement of risk stratification.	- Evidence currently limited to very few studies.
CARDIAC SARCOIDOSIS (CS)	- Detection of subtle patterns of myocardial inflammation and fibrosis; - Quantitative analysis may also support assessment of disease activity.	- Small sample sizes and heterogeneous methodologies.

**Table 3:** Characteristics and main findings of included studies.

First Author, Year	Disease Category	*N* Patients	Analysis	Main Findings
Amano et al., 2020 [5]	HCM	31 (HCM 20; controls 11)	Texture analysis of T2-weighted STIR images to detect myocardial tissue alterations in HCM.	Texture features identified myocardial tissue abnormalities in HCM that were not detectable using conventional T2 mapping alone.
Antonopoulos et al., 2021 [6]	HCM/CA	149 (HCM 61; CA 28; LVH 30; controls 30)	Machine-learning analysis of native T1 mapping-derived radiomic features to classify cardiac disease phenotypes including HCM and CA.	T1-derived radiomic signatures improved disease classification and identification of HCM phenocopies compared with native T1 values alone.
El-Rewaidy et al., 2020 [7]	HCM/IDCM	321 (HCM 116; DCM 140; controls 65)	Texture analysis of native T1 mapping to characterize myocardial fibrosis patterns in HCM, DCM, and controls.	Native T1 texture identified disease-specific fibrosis patterns and provided additional information beyond global native T1 values.
Liu et al., 2023 [8]	HCM	345	CMR radiomics of the myocardium and papillary muscles to detect LVH and differentiate HCM from HHD.	Myocardial and papillary muscle radiomic features enabled LVH detection and HCM/HHD differentiation, with the combined MYO + PM model showing the best performance.
Neisius et al., 2019 [9]	HCM	232 (HCM 108; HHD 53; controls 71)	Native T1 mapping-based texture analysis to differentiate HCM from HHD.	Selected T1 texture features differentiated HCM from HHD with high diagnostic accuracy and outperformed global native T1 values.
Neisius et al., 2020 [10]	HCM	217 (HCM 188; controls 29)	Native T1 mapping-based texture analysis to identify HCM patients with myocardial scar on LGE imaging.	A radiomic model predicted LGE positivity and identified a subset of LGE-negative patients, suggesting a potential role for reducing gadolinium administration in selected cases.
Schofield et al., 2018 [11]	HCM/CA	216 (HCM 50; CA 52; AS 68; HHD 15; controls 31)	Cine-CMR texture analysis to differentiate etiologies of LVH, including HCM and CA.	Cine-derived texture features differentiated LVH etiologies, with HCM and CA showing the greatest deviation from healthy myocardium.
Wang et al., 2020 [12]	HCM	102 (MYH7 68; MYBPC3 34)	Native T1 mapping radiomics to differentiate MYH7-related from MYBPC3-related HCM.	Radiomic features identified genotype-related phenotypic differences that were not captured by conventional native T1 measurements.
Wang et al., 2021 [13]	HCM	379	LGE-CMR radiomics to evaluate prognostic markers for SCD risk in HCM.	LGE-derived radiomic features were associated with SCD events; LBP-19 and Moment-1 were independent predictors after adjustment for clinical variables.
Zhang et al., 2024 [14]	HCM	621 (HCM 421; HHD 200)	Cine- and LGE-CMR radiomics combined with conventional CMR parameters to differentiate HCM from HHD.	A combined model integrating conventional CMR parameters, cine radscore, and LGE radscore showed excellent discrimination between HCM and HHD, outperforming conventional CMR parameters alone.
Zhang et al., 2025 [15]	CA/HCM	253 (AL-CA 121; HCM 84; controls 48)	Multiparametric CMR radiomics with ensemble machine learning to differentiate AL-CA from HCM and controls.	The ensemble model achieved high multiclass diagnostic performance for AL-CA, HCM, and controls, with mapping-derived texture and first-order features contributing substantially to discrimination.
Zhang et al., 2023 [16]	HCM/IDCM	283 (HCM 68; DCM 72; controls 143)	CMR radiomics with machine learning to classify HCM, DCM, and normal myocardium.	A radiomics model based on selected features achieved high classification accuracy for HCM, DCM, and controls.
Amano et al., 2025 [17]	IDCM	47	Non-contrast CMR, including cine imaging, T1/T2 mapping, and T1 texture analysis, to identify heart failure etiologies including DCM.	Native T1 values and T1 texture features helped differentiate DCM from other heart failure etiologies, while cine parameters supported differentiation of DCM from tachycardia-induced cardiomyopathy.
Amyar et al., 2025 [18]	IDCM	132	Radiomic features extracted from multiparametric CMR sequences (T1 mapping, ECV, and LGE) analyzed in relation to myocardial histology in non-ischemic DCM.	CMR radiomic features were associated with myocardial histological findings. Texture features from T1, ECV, and LGE reflected underlying tissue changes such as fibrosis, inflammation, myocyte hypertrophy, and fat replacement.
Deng et al., 2024 [19]	IDCM	115 (ICM 64; DCM 51)	Cine-CMR radiomics combined with machine learning to differentiate ICM from DCM.	Cine-derived radiomics improved differentiation between ICM and DCM compared with clinical features alone, supporting a potential role for non-contrast etiological classification.
Izquierdo et al., 2021 [20]	IDCM/HCM	118 (LVNC 35; HCM 25; DCM 37; controls 21)	Cine-CMR radiomics with machine learning to differentiate LVNC, HCM, DCM, and healthy controls.	Cine-derived radiomics enabled automated differentiation of LVNC, HCM, DCM, and controls, with performance comparable to conventional CMR indices.
Lasode et al., 2025 [21]	IDCM	200	Non-contrast cine-CMR radiomics to differentiate ICM from DCM and detect myocardial scar.	Cine-CMR radiomics enabled ICM/DCM differentiation and scar detection without contrast administration, suggesting potential value when gadolinium is contraindicated.
Shao et al., 2018 [3]	IDCM	74 (DCM 50; controls 24)	Texture analysis of native T1 mapping images using histogram and gray-level co-occurrence matrix features with machine learning classification.	Texture features extracted from T1 maps identified myocardial tissue alterations and enabled differentiation between DCM patients and healthy controls.
Huang et al., 2022 [22]	CA/HCM	317 (CA 100; HCM 217)	T2-weighted CMR texture analysis to differentiate CA from HCM.	T2-weighted texture features captured myocardial tissue differences between CA and HCM and showed discriminatory potential comparable to LGE-based assessment.
Jiang et al., 2022 [23]	CA/HCM	251 (CA 85; HCM 82; controls 84)	Non-contrast cine-CMR radiomics combined with machine learning to differentiate CA from HCM.	Cine-derived radiomics improved discrimination between CA and HCM compared with conventional CMR parameters; GLevNonU was the most discriminative feature.
Ma et al., 2024 [24]	CA	114 (suspected AL-CA 86; controls 28)	Native T1 mapping-derived radiomics to detect myocardial involvement in AL-CA.	A native T1 radiomics score improved detection of myocardial involvement in AL-CA compared with native T1 measurements alone.
Zhou et al., 2022 [25]	CA	200	LGE-CMR radiomics with machine learning to diagnose CA in a multicohort diagnostic study.	LGE-derived radiomics provided incremental diagnostic information over visual assessment and quantitative CMR parameters, with radiomic scores reflecting disease severity.
Guo et al., 2025 [26]	ARCV	122	Pericardial fat radiomics from cine-CMR with automated segmentation to predict LV involvement and outcomes in ARVC.	Pericardial fat radiomic features predicted LV involvement and were associated with adverse cardiovascular outcomes, providing incremental prognostic information in ARVC.
Le et al., 2025 [27]	CS	108 (CS 75; NICM 33)	LGE-CMR radiomics to quantitatively characterize CS and differentiate it from other NICM phenotypes.	LGE-derived radiomic patterns characterized CS-related myocardial injury and supported differentiation from other NICM phenotypes.
Mushari et al., 2023 [28]	CS	61 (active CS 30; inactive CS 31)	LGE-CMR radiomics to differentiate active from inactive CS using PET-CMR-defined disease activity as reference.	CMR radiomic features showed potential to differentiate active from inactive CS, supporting their use as quantitative markers of disease activity.
Mushari et al., 2024 [29]	CS	75 (CS 40; post-COVID 35)	PET and LGE-CMR radiomics to identify CS and differentiate it from post-COVID myocardial inflammation.	PET and CMR radiomic features showed potential for distinguishing CS from other inflammatory myocardial conditions, supporting radiomics as a quantitative adjunct to conventional imaging.

Abbreviations: AL-CA: amyloid light-chain cardiac amyloidosis; ARVC: arrhythmogenic right ventricular cardiomyopathy; AS: aortic stenosis; CA: cardiac amyloidosis; CMR: cardiovascular magnetic resonance; CS: cardiac sarcoidosis; DCM: dilated cardiomyopathy; ECV: extracellular volume; GLevNonU: gray-level non-uniformity; HCM: hypertrophic cardiomyopathy; HHD: hypertensive heart disease; ICM: ischemic cardiomyopathy; IDCM: idiopathic dilated cardiomyopathy; LBP: local binary pattern; LGE: late gadolinium enhancement; LV: left ventricular; LVH: left ventricular hypertrophy; LVNC: left ventricular non-compaction; MYO: myocardium; NICM: non-ischemic cardiomyopathy; PET: positron emission tomography; PM: papillary muscle; SCD: sudden cardiac death; STIR: short tau inversion recovery.

## Data Availability

No new data were created or analyzed in this study. Data sharing is not applicable to this article.
